# Risk Factors Influencing Mortality in Open Necrosectomy for Acute Pancreatitis: A Comparative Analysis

**DOI:** 10.3390/jcm13237151

**Published:** 2024-11-26

**Authors:** Tudorel Mihoc, Catalin Pirvu, Amadeus Dobrescu, Dan Brebu, Anca Monica Oprescu Macovei, Zoran Laurentiu Popa, Stelian Pantea

**Affiliations:** 1 Department X, Surgical Emergencies Clinic, “Victor Babeș” University of Medicine and Pharmacy Timișoara, Romania, 300041 Timișoara, Romania; mihoc.tudorel@umft.ro (T.M.); pirvu.catalin@umft.ro (C.P.); pantea.stelian@umft.ro (S.P.); 2 Department X, 2nd Surgical Clinic, Researching Future “Chirurgie 2”, “Victor Babeș” University of Medicine and Pharmacy Timișoara, Romania, 300041 Timișoara, Romania; dobrescu.amadeus@umft.ro (A.D.); brebu.dan@umft.ro (D.B.); 3 Department of Gastroenterology, Emergency Hospital Prof. Dr. Agripa Ionescu, “Carol Davila” University of Medicine and Pharmacy, 050474 Bucuresti, Romania; anka_makovei@yahoo.com; 4 Department of Obstetrics and Gynecology, “Victor Babeș” University of Medicine and Pharmacy Timișoara, Romania, 300041 Timișoara, Romania

**Keywords:** pancreatic necrosis, acute necrotic collection, walled-off necrosis, open necrosectomy

## Abstract

**Background and Objectives**: Patients undergoing open necrosectomy (ON) for acute pancreatitis (AP) often face high morbidity and mortality rates. This study aims to identify risk factors associated with adverse outcomes by comparing survivors and non-survivors of ON. **Materials and Methods**: A retrospective analysis was conducted on 74 patients who underwent ON for AP. Patients were divided into two groups: survivors (n = 52) and non-survivors (n = 22). Preoperative and postoperative variables were compared, and *p*-values were calculated to identify significant differences. **Results**: The mortality rate was 29.73%. Significant risk factors for mortality included age over 60 (*p* = 0.008), multiple organ failure (*p* = 0.001), early necrosectomy before 28 days (*p* = 0.001), higher neutrophil-to-lymphocyte ratio (NLR) (*p* = 0.045), and postoperative complications such as bleeding (*p* = 0.005) and intestinal fistula (*p* = 0.038). Delayed ON after 28 days showed a significantly lower mortality rate (12.5%) compared to early ON (50%). **Conclusions**: Age, severity of AP, timing of ON, and postoperative complications significantly influence mortality rates in patients undergoing ON. Delaying surgery beyond 28 days and optimizing surgical techniques may improve patient outcomes.

## 1. Introduction

Patients with acute pancreatitis (AP) consume significant financial resources of health services all over the world. One in every five patients with AP will develop a complication, such as the necrosis of the pancreatic or peripancreatic tissues [1], with mortality rates that could rise up to 39% [2]. Two main factors were found responsible for these alarming death rates in patients with complicated AP: the uncontrollable inflammatory systemic response (SIRS), which leads to multiple organ and system failures (MSOFs), and infected necrosis with consecutive sepsis, SIRS, and MSOF [3].

According to the revised Atlanta Classification, there are two types of necrosis in the evolution of complicated acute pancreatitis: acute necrotic collection (ANC) and walled-off necrosis (WON) [1]. ANC is present in the first four weeks of AP evolution and has no wall, while WON characterizes AP after four weeks from the debut and has a well-defined inflammatory wall. Both types of necrosis can get infected, triggering different grades of inflammatory responses from leukocytosis to sepsis and death.

In the surgical management of acute pancreatitis complicated by necrosis, various strategies have evolved to address the severity and outcomes associated with this condition. Traditionally, infected necroses were treated with necrosectomy by means of open surgery, leading to high morbidity and mortality rates [4]; nowadays, better defined step-up treatment is used, which includes a conservative medical treatment, percutaneous or transgastric drainage, minimally invasive necrosectomy, and only in selected cases or in cases where the above algorithm fails, the surgeon performs an open necrosectomy [5,6,7]. Mortality up to 22.9% was described in a highly specialized center after ON in AP [8]; nonetheless, ON must remain a strong option for surgeons due to unpredictability of AP course. Open necrosectomy has a status of last resort procedure against infected peripancreatic necrosis or as a saving procedure for complications due to the minimally invasive procedures [6].

In our tertiary hospital, we mainly performed open necrosectomies, through which we gained important experience, and minimally invasive techniques for pancreatic necrosectomy had a slow start. Despite our surgical expertise, the rate of success in treating these patients was influenced by the patient’s age and AP complications. Therefore, the aim of this study was to present the morbidity and mortality rates of a retrospective cohort of AP treated with ON in our hospital and to find the main risks factors for these unfavorable outcomes.

## 2. Materials and Methods

### 2.1. Patients’ Population

A retrospective study was performed on the patients with acute pancreatitis who underwent open necrosectomy between 1 January 2020 and 31 December 2023. The study was approved by the hospital ethics committee that follows Helsinki guidelines for research ethics.

The selection of the patients was conducted by using the following searching methods: ICD-10 diagnostic and surgical procedure codes were applied as filters on the hospital patient’s electronic database. The study on the effectiveness of open necrosectomy in treating acute pancreatitis (AP) at a tertiary hospital meticulously defined its inclusion and exclusion criteria to ensure the reliability and specificity of its findings. The inclusion criteria included the following: (1) patients diagnosed with AP between 1 January 2020 and 31 December 2023, who underwent ON; (2) those identified using ICD-10 diagnostic codes specific to AP and corresponding surgical procedure codes; (3) patients whose conditions involved microbiologically confirmed infected necrosis; and (4) AP patients with a prolonged hospital stay, identified through manual file review if initial electronic searches were inconclusive.

Conversely, the exclusion criteria were comprehensive to isolate the impact of ON: (1) patients who underwent the index surgical procedure at a facility other than the study hospital; (2) patients treated with any minimally invasive necrosectomy techniques; (3) those who developed abdominal compartment syndrome prior to the index necrosectomy; (4) patients with incomplete data on the surgical procedure or subsequent complications; and (5) patients diagnosed with COVID-19 during the later phase of the study period. Follow-up was 90 days after discharge, with patient survival as the primary outcome.

### 2.2. Data Management

Organ failure was assessed according to the Marshal criteria and was considered as persistent if it lasted for more than 48 h. Those patients who had AP with organ failures but died in less than 48 h from surgery were considered to have had a persistent organ failure. If a patient had persistent organ failure concomitant with a transient organ failure, the patient was included in the single persistent organ failure group.

CT scan severity index (CTSI) was calculated according to the Balthazar score [9] and the extent of pancreatic necrosis. Radiologic images were reviewed and compared with the original results by the surgeons involved in the study with the help of a senior radiologist when those images were present in the hospital electronic archive.

The following patients’ demographics and clinical variables were extracted and recorded: gender, age, etiology, organ failure, CTSI, lipase, neutrophils–lymphocytes ratio, platelets–lymphocytes ratio, CRP, form of the AP, indication for surgery, the surgical procedures and related complications, infection of the necrosis, hospital stay, days in the ICU, and death. All laboratory parameters were measured at admission.

The diagnosis of infected necrosis was based upon positive aspiration cultures from the necrotic collection, a CT scan revealing gas bubbles present in the collection, or clinical signs of sepsis and a failure to thrive of the patient established through daily clinical observation. The definitive diagnosis of infected necrosis was acknowledged after surgery. The decision for surgery was based on medical treatment failure.

Surgical procedures were standard: an anterior abdominal midline incision was performed and the approach of pancreatic and peripancreatic necrosis was performed through the lesser sac. For distant necrosis involving the root of the mesentery, the inframesocolic route was used, and for retrocolic necrosis, lateral colonic mobilizations were performed for each side involved, followed by necrosectomy. Blunt necrosectomy was performed by hand or suction to avoid damaging the large blood vessels and excessive bleeding. Packing and drainage or just drainage was the next step, and whenever possible, the retroperitoneal route was used for exteriorizing up to four drains.

In our study, we considered both the traditional open necrosectomy and the modified minimally invasive necrosectomy to treat necrotizing pancreatitis. Previously, open necrosectomy involved a large abdominal incision to remove necrotic pancreatic tissue, which often led to significant morbidity. To improve outcomes, we also explored the minimally invasive approach, which utilized smaller incisions or natural orifices along with endoscopic tools, aiming to reduce the invasiveness of the procedure. This method was anticipated to offer benefits such as reduced pain, faster recovery, and shorter hospital stays.

### 2.3. Statistical Analysis

The statistical analysis was performed using MedCalc Version 19.3.1. Descriptive statistics were used for clinical, anthropometric, and demographic data of the patients. Numerical variables with normal distribution were presented as the means ± standard deviation, while variables with non-normal distribution were presented as median values and range. The Kolmogorov–Smirnov test was used for measuring the distribution of numerical variables. Qualitative variables were presented as numbers and percentages. Parametric tests (*t*-test) were used for the assessment of differences between numerical variables with normal distribution and nonparametric tests (Mann–Whitney or Kruskal–Wallis tests) for variables with non-normal distribution. The Chi-square (X2) test (with Yates’ correction for continuity) was used for comparing proportions expressed as percentages (“n” designates the total number of subjects from a particular group). Confidence intervals (95%) were calculated for each predictive test and a *p*-value < 0.05 was considered as significant for each statistical test. The Pearson test was used for correlations.

The statistical power was calculated based on assumptions derived from preliminary data and similar studies. The power was set at 0.80, and the alpha level was established at 0.05, which are conventional standards designed to detect a true effect when present. The effect size was determined from clinical importance and corroborated by prior research, with a moderate level set (Cohen’s d = 0.5). Given these parameters, the power calculation indicated that a sample size of 64 participants (32 per group) was required to achieve sufficient statistical power to detect the specified effect size with the designated level of significance.

## 3. Results

There were 123 patients identified with AP and necrosectomy in the established searching interval, from which 74 patients were included in the study. The reasons for the 49 excluded were as follows: 20 patients had abdominal compartment syndrome, 18 patients had minimally invasive procedures, 8 patients already had a necrosectomy in another hospital before being transferred, 2 patients died because of infection with SARS-CoV-2, and 1 patient did not have clear information regarding the surgical procedure. Preoperative demographics, and clinical, biological, and radiologic findings are listed in Table 1.

In Table 1, we compare preoperative clinical and demographic variables between survivors and non-survivors. The median age was significantly higher in non-survivors (65 years) compared to survivors (48 years), with a *p*-value of 0.008, indicating age as a significant risk factor for mortality. Multiple organ failure before surgery was significantly more prevalent in non-survivors (81.82%) compared to survivors (50.00%), with a *p*-value of 0.009. Additionally, early necrosectomy before 28 days was performed more frequently in non-survivors (81.82%) than survivors (28.85%), suggesting that delayed surgery might improve outcomes.

Table 2 highlights postoperative variables. Non-survivors had a significantly higher rate of postoperative bleeding (22.73%) compared to survivors (1.92%), with a *p*-value of 0.005. Intestinal fistula occurrence was also higher in non-survivors (22.73%) than in survivors (5.77%), indicating these complications as significant risk factors. Persistent new organ failure post-surgery was significantly more common in non-survivors (31.82%) compared to survivors (3.85%), with a *p*-value of 0.001. These findings suggest that postoperative management should focus on preventing complications to improve survival rates.

Table 3 presents laboratory findings. Non-survivors had significantly higher white blood cell counts (*p* = 0.012) and serum creatinine levels (*p* = 0.004), indicating severe infection and renal impairment. Platelet counts were lower in non-survivors (*p* = 0.047), which might be associated with coagulopathy or consumption due to sepsis. Elevated blood urea nitrogen levels in non-survivors (*p* = 0.009) further support the presence of renal dysfunction. These laboratory parameters can serve as important prognostic indicators in patients undergoing ON.

**Figure 1:**
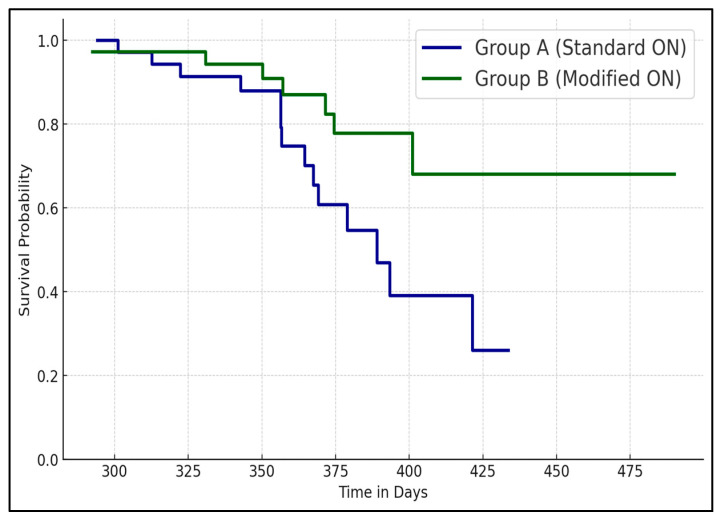
Kaplan–Meier survival analysis based on ON type.

Table 4 and Figure 1 compare outcomes between standard ON (Group A) and modified ON techniques (Group B). Group B had a significantly shorter surgery duration (*p* = 0.002) and less blood loss (*p* = 0.005), suggesting that the modified technique may be more efficient. Although not statistically significant, Group B showed lower rates of postoperative bleeding and infection. Mortality rates were slightly lower in Group B but did not reach statistical significance (*p* = 0.627), indicating that while the modified technique may reduce some complications, it may not significantly impact mortality.

Table 5 examines the length of hospital and ICU stay. Non-survivors tended to have longer ICU stays and more days on mechanical ventilation, with ventilator days being significantly higher (*p* = 0.018). This suggests that prolonged respiratory support is associated with higher mortality. While the total length of hospital stay and total nutrition days were longer in non-survivors, these differences were not statistically significant. These findings highlight the importance of optimizing respiratory care in critically ill patients.

Table 6 explores the impact of comorbidities. Chronic kidney disease was significantly more common in non-survivors (27.27%) compared to survivors (5.77%), with a *p*-value of 0.014. This indicates renal impairment as a significant risk factor for mortality. Other comorbidities like diabetes mellitus, cardiovascular disease, obesity, and chronic lung disease were more prevalent in non-survivors but did not reach statistical significance. This suggests that while these conditions may contribute to overall risk, their individual impact may be less pronounced.

The logistic regression analysis presented in Table 7 evaluated the impact of various risk factors on mortality among patients undergoing open necrosectomy, with data categorized into survivors and non-survivors. The results indicated significant associations between several clinical factors and increased mortality risk. Specifically, age greater than 60 years, early necrosectomy (within 28 days), multiple organ failure, and chronic kidney disease significantly correlated with higher mortality rates, as evidenced by *p*-values of 0.003 or less. The strongest predictor among these was multiple organ failure, which was associated with over a fourfold increase in mortality risk (OR = 4.6, 95% CI: 2.3–9.1). The findings also showed that a high neutrophil-to-lymphocyte ratio (NLR > 15) and the presence of an intestinal fistula modestly increased mortality risks, with respective odds ratios of 1.9 and 2.1. In contrast, postoperative bleeding, although presenting an elevated odds ratio, did not statistically influence mortality outcomes (*p* = 0.108). These insights highlighted critical areas for clinical focus, particularly the management of patients with identifiable high-risk factors, to potentially improve survival rates following open necrosectomy.

## 4. Discussion

The current study showed that open necrosectomy has its role in the treatment of AP that does not respond to conservative treatment. By comparing the survivors with the non-survivors, we could identify the following differences: early ON prompted by adverse events in the disease’s course had a worse outcome, a death rate five times higher due to multiple OF, more postoperative new organ failures, due to postoperative surgical complications. Husu et al. found that the factors directly related with patients’ deaths were age over 60 years, pre-existing comorbidities, early necrosectomy within the first 28 days, multiple OF, white blood cell (WBC) count over 23.000/µL, and deterioration or prolonged organ failure, while patients having none of these factors had no mortality [8].

The only modifiable factors influencing mortality in our study were the morbidities of the AP, the timing of the procedure, and the number of the procedures. The complications of an AP are hard to predict and manage, but intensive care or intermediate care units should be involved when treating these patients [6]. The timing of the procedure could be delayed in order to lower the mortality, but a patient who is non-responsive to conservative treatment will become more ill; unfortunately, there are no studies trying to find these sub-group of patients who need surgery for early complications, and to search for the best timing of the procedure for them. The number of procedures could be influenced by the surgeons using proper techniques, but even when highly specialized surgeons were in charge, half of the patients required reoperation [8].

The death rate of late ON was comparable between our study and theirs, but overall mortality rate was higher in our study, most likely due to the higher percentage of severe forms of AP with persistent OF. Others found very low rates of mortality, but they achieved this by mixing a higher percentage of patients with MSAP in the analyzed pool. Madenci et al. reported a 6% death rate, but included only 35% SAP patients [10], while Maatman et al. reported a 1.7% death rate, with an unreported proportion of SAP, most likely below 30% [11].

On the other side of the table, Buasch et al. reported a 69% death rate after ON with 93% of the patients with sepsis [12]. Sgaramella et al. pointed out in a review that studies were heterogenous when reporting data, with many missing important information like the AP form classified according to the revised Atlanta 2012 classification, the reporting of comorbidities, and even the description of the collection; additionally, 70% of the studies reported CTSI [13].

The fact that ON performed on patients with MSAP had no mortality was a strong argument for exercising caution with the procedure and stating that the most important factors related to the death of the patients were the age and comorbidities or the morbidities associated with the AP.

Minimally invasive techniques for the debridement of the necrotic pancreatic and peripancreatic tissues were developed at the beginning of the millennium, starting with percutaneous retroperitoneal necrosectomy with the nephroscope, endoscopic transluminal necrosectomy from the stomach or duodenum, or the video-assisted retroperitoneal debridement approach [14,15,16]. Despite advances in scope technology and in the training of doctors using these tools, patients requiring a rapid solution are still referred to ON. Nonetheless, some patients are suitable for a step-up approach, starting with a minimally invasive procedure, as recommended by Besselink: “delay, drain, debride” [17,18,19].

Most of the studies comparing these different approaches were either retrospective or prospective studies; van Santoort et al. published a randomized control trial where minimally invasive techniques were viewed as the better options, despite having the same mortality rate with ON, but with fewer major complications [7]. The biggest argument in favor of keeping ON in the surgical arsenal is that ON is a bailed-out procedure for all sort of complications of minimally invasive techniques [8].

Our study brought NLR as a new risk factor for mortality. NLR is regarded as a cheap and fast marker of inflammation, and its value for the AP prognostic has been evaluated in many studies [20]. In our study, an elevated value was an indicator of a higher risk of death, but the relatively small number of patients in this study could be a source of error, and we did not try to find a cut-off value.

The findings of this study underscore critical risk factors influencing mortality in patients undergoing open necrosectomy for acute pancreatitis. Recent advancements in endoscopic ultrasound (EUS) have demonstrated significant utility in the differential diagnosis and management of pancreatic conditions, as noted by Boicean et al. (2023) [21]. EUS allows for high-resolution imaging of pancreatic necrosis and associated complications, providing detailed assessment of tissue characteristics that could guide clinical decisions on surgical timing. Advanced techniques, such as contrast-enhanced EUS and elastography, further enhance diagnostic accuracy by evaluating perfusion and stiffness, enabling more informed management strategies. Incorporating these modalities into preoperative planning for ON may help optimize patient selection and avoid premature interventions, potentially reducing mortality rates associated with early necrosectomy.

Emerging therapeutic approaches, such as fecal microbiota transplantation (FMT), could also play a pivotal role in the management of AP. Alterations in gut microbiota have been implicated in the progression of AP, particularly in severe cases involving systemic inflammation and multiple organ failure. One study suggests that FMT offers a promising strategy for restoring microbial balance and reducing inflammatory responses, showing potential to improve outcomes in AP [22]. While this study highlights the importance of surgical timing and optimization, integrating FMT into the treatment paradigm may complement surgical strategies by mitigating systemic complications. Future research should investigate the interplay between gut microbiota and surgical outcomes, exploring whether combining FMT with delayed ON could further improve survival rates in patients with severe AP.

A potential limitation of this study is the variation in surgical teams performing the procedures, as differences in technique and experience among surgeons could have influenced the outcomes observed between Group A and Group B. One other possible flaw of our study is that most of the procedures were performed as an emergency operation by surgeons who were on call, not by specialized surgeons (86% cases), as in the study of Husu et al. [8], a fact that could also explain our higher death rate of 29% vs. 22%. Husu et al. reported a 10% pancreatic resection rate at the indexed necrosectomy, while we observed a 3.84% pancreatic resection rate after a longer period after first necrosectomy. The removal of the necrotic tail/and or the body of the pancreas during the indexed necrosectomy could account for the different numbers. In contrast, van Santvoort reported more than 30% pancreatic fistula but no pancreatic resection [7,8].

## 5. Conclusions

This retrospective study underscores that advanced age, multiple organ failure, and chronic kidney disease are critical risk factors significantly associated with increased mortality in patients with acute pancreatitis undergoing open necrosectomy. Additionally, performing necrosectomy earlier than 28 days post-diagnosis was linked to poorer survival outcomes, highlighting the potential benefits of delayed surgical intervention. Postoperative complications, particularly persistent organ failure and intestinal fistulas, further exacerbated mortality risks, emphasizing the necessity for meticulous postoperative management. While the adoption of modified minimally invasive necrosectomy techniques reduced surgery duration and blood loss, it did not translate into a statistically significant decrease in mortality rates. These findings reflect the complex interplay between patient-related factors and surgical outcomes, suggesting that optimizing patient selection and timing for ON, alongside enhanced management of comorbidities and postoperative complications, are essential strategies for improving survival rates in AP patients treated with open necrosectomy.

## Figures and Tables

**Table 1:** Preoperative clinical and demographic variables of patients.

Variables	Survivors (n = 52)	Non-Survivors (n = 22)	*p*-Value
Age, Median (IQR), Years	48 (40–59)	65 (58–70)	**0.008** *
Male	38 (73.08%)	18 (81.82%)	0.412 **
Etiology			
-Biliary	24 (46.15%)	10 (45.45%)	0.952 **
Alcohol	17 (32.69%)	6 (27.27%)	0.647 **
Hypertriglyceridemia	3 (5.77%)	1 (4.55%)	0.831 **
Others/Idiopathic	8 (15.38%)	5 (22.73%)	0.422 **
AP Form			**0.041** **
-Severe AP	35 (67.31%)	20 (90.91%)	
Moderate Severe AP	17 (32.69%)	2 (9.09%)	
Organ Failure Before Surgery			
-No Organ Failure	3 (5.77%)	0 (0%)	0.255 **
-Single Organ Failure	23 (44.23%)	4 (18.18%)	**0.037** **
-Multiple Organ Failure	26 (50.00%)	18 (81.82%)	**0.009** **
CRP Before Surgery (mg/L)	210 (75–350)	230 (80–370)	0.672 **
NLR Before Surgery	10.5 (7.5–12.5)	14.8 (9.5–18.0)	**0.045** **
Necrosectomy Timing			**0.001** **
Before 28 days	15 (28.85%)	18 (81.82%)	
After 28 days	37 (71.15%)	4 (18.18%)	

*—Mann–Whitney test; **—Chi-square or Fisher’s test; AP—acute pancreatitis; SAP—severe acute pancreatitis; MSAP—moderate severe acute pancreatitis; CRP—C reactive protein; FNA—fine needle aspiration; CT scan—computer tomography.

**Table 2:** Postoperative variables of patients.

Variables	Survivors (n = 52)	Non-Survivors (n = 22)	*p*-Value
Infected Necrosis	40 (76.92%)	21 (95.45%)	0.071 **
Reoperation			
One Necrosectomy	30 (57.69%)	8 (36.36%)	0.096 **
Two Necrosectomies	12 (23.08%)	5 (22.73%)	0.971 **
More than Two Necrosectomies	4 (7.69%)	6 (27.27%)	**0.031** **
Postoperative Bleeding	1 (1.92%)	5 (22.73%)	**0.005** **
Intestinal Fistula	3 (5.77%)	5 (22.73%)	**0.038** **
New Organ Failure Post-Surgery		20 (90.91%)	**0.041** **
Transient	4 (7.69%)	0 (0%)	0.18 **
-Persistent	2 (3.85%)	7 (31.82%)	**0.001** **
Length of ICU Stay (days)	15 (5–25)	18 (7–30)	0.212 *
Death	0 (0%)	22 (100%)	N/A

*—Mann–Whitney test; **—Chi-square or Fisher’s test; ICU—intensive care unit.

**Table 3:** Laboratory findings of patients.

Variables	Survivors (n = 52)	Non-Survivors (n = 22)	*p*-Value *
White Blood Cells (×10^9^/L)	13.5 (9.0–16.0)	17.8 (14.5–22.3)	**0.012**
Platelets (×10^9^/L)	220 (180–260)	190 (150–230)	**0.047**
Serum Creatinine (μmol/L)	90 (70–110)	130 (100–160)	**0.004**
Blood Urea Nitrogen (mmol/L)	6.5 (5.0–8.0)	9.2 (7.5–11.0)	**0.009**
Serum Lipase (U/L)	400 (250–550)	350 (200–500)	0.325

*—Mann–Whitney test.

**Table 4:** Comparison of surgical techniques.

Variables	Group A (Standard ON, n = 37)	Group B (Modified ON, n = 37)	*p*-Value
Average Surgery Duration (min)	150 (130–170)	130 (110–150)	0.002 *
Blood Loss (mL)	500 (400–600)	400 (300–500)	0.005 *
Postoperative Complications			
- Bleeding	6 (16.22%)	2 (5.41%)	0.138 **
- Infection	10 (27.03%)	5 (13.51%)	0.155 **
Mortality Rate	12 (32.43%)	10 (27.03%)	0.627 **

*—Mann–Whitney test; **—Chi-square or Fisher’s test.

**Table 5:** Length of hospital and ICU stay.

Variables	Survivors (n = 52)	Non-Survivors (n = 22)	*p*-Value *
Length of Hospital Stay (days)	30 (20–40)	25 (15–35)	0.067
Length of ICU Stay (days)	15 (5–25)	18 (7–30)	0.212
Ventilator Days	10 (5–15)	15 (10–20)	0.018
Total Nutrition Days	20 (15–25)	22 (18–28)	0.089

ICU—intensive care unit; *—Mann–Whitney test.

**Table 6:** Comorbidities and their impact on outcomes.

Variables	Survivors (n = 52)	Non-Survivors (n = 22)	*p*-Value **
Diabetes Mellitus	10 (19.23%)	8 (36.36%)	0.108
Cardiovascular Disease	15 (28.85%)	10 (45.45%)	0.16
Chronic Kidney Disease	3 (5.77%)	6 (27.27%)	0.014
Obesity (BMI > 30 kg/m^2^)	12 (23.08%)	9 (40.91%)	0.121
Chronic Lung Disease	5 (9.62%)	4 (18.18%)	0.303

BMI—Body Mass Index; **—Chi-square or Fisher’s test.

**Table 7:** Univariate logistic regression analysis for risk factors influencing mortality in open necrosectomy.

Variables	OR	95% CI	*p*-Value
Age > 60 years	2.8	1.4–5.6	0.003
Early Necrosectomy (<28 days)	3.2	1.8–5.7	0.001
Multiple Organ Failure	4.6	2.3–9.1	0.001
NLR > 15	1.9	1.1–3.2	0.024
Postoperative Bleeding	1.7	0.9–5.4	0.108
Intestinal Fistula	2.1	1.0–4.3	0.046
Chronic Kidney Disease	3.3	1.5–7.2	0.003
Severe AP Form (vs. Moderate)	2.7	1.3–5.4	0.008

## Data Availability

Data availability is subject to the hospital’s approval.
